# Subcutaneous soft tissue metastases from esophageal squamous cell carcinoma with neuroendocrine differentiation: Case report and literature review

**DOI:** 10.3389/fonc.2022.895189

**Published:** 2022-08-12

**Authors:** Xiaotao Geng, Jie Liu, Huimin Sun, Zhenguo Song, Shaoyong Qin, Yang Li, Yanan Zhang, Furong Hao, Yuanyuan Cai

**Affiliations:** ^1^ Department of Radiation Oncology, Weifang People’s Hospital, Weifang, China; ^2^ Department of Pathology, Weifang People’s Hospital, Weifang, China; ^3^ Department of Nuclear Medicine, Weifang People’s Hospital, Weifang, China; ^4^ Department of Ultrasound, Weifang People’s Hospital, Weifang, China

**Keywords:** esophagus, esophageal carcinoma, squamous cell carcinoma, subcutaneous soft tissue metastasis, soft tissue metastasis, radiotherapy, chemotherapy, immunotherapy

## Abstract

**Background:**

Esophageal squamous cell carcinoma is the predominant subtype of esophageal cancer in China and so differs from presentations in Western countries. Common metastatic locations of esophageal cancer include the liver, lung, bone, and brain. In contrast, metastases in subcutaneous soft tissue are exceedingly rare.

**Case presentation:**

We present the experience of a 57-year-old man with a complaint of hand and leg dysfunction on the right side. He had a past medical history of esophageal squamous cell carcinoma. Further imaging workup revealed a solitary brain metastasis, thickening of the esophageal wall, swollen lymph nodes in the mediastinum, and right adrenal gland metastasis. Gamma knife radiosurgery of the brain metastasis and intensity-modulated radiotherapy of the esophagus and lymph nodes were administered. After 1.5 months, he was admitted to our hospital again, and nodules were identified in the anterior abdominal wall and left posterior chest wall. Ultrasound, CT, and radical excision of the abdominal wall mass were undertaken and revealed metastatic squamous cell carcinoma with neuroendocrine differentiation. We administered immunotherapy followed by targeted therapy. A PET/CT scan was performed to identify other organ metastases; the scan revealed multiple areas of fluorodeoxyglucose uptake and foci in the esophagus, lung, liver, bone, and right adrenal gland; and in various lymph nodes. In addition, an intensely hypermetabolic lesion was localized in the left posterior thorax.

**Conclusion:**

This case highlights the diagnosis and treatment of uncommon metastases of esophageal squamous cell carcinoma. We hope that our clinical experience provides insights into these uncommon metastases.

## Introduction

Esophageal cancer (EC) is the eighth most common cancer and the sixth most common cause of cancer-related mortality worldwide; in 2020, 604,100 new cases and 544,076 new deaths were recorded (1). The incidence and mortality of ES are more severe in China, where it is the sixth most common cancer and the fourth leading cause of cancer-related death, than in Western countries (2). Esophageal squamous cell carcinoma (ESCC) is the major type of EC in Asia; it accounts for 90% of ECs in the Asian population and differs from the presentation in Western countries (3). Although improvements in treatment *via* immunotherapy and targeted therapy have been achieved in recent years, the 5-year survival rate of EC remains poor (4). Locoregional recurrence and distant metastases remain the main failure patterns of ESCC after treatment (5, 6). According to a population-based study of EC, the liver is the most common metastatic organ, followed by the lung, bone, and brain (7). Here, we report one patient with subcutaneous soft tissue metastases after initial treatment.

## Case presentation

A 57-year-old Chinese man with a past medical history of ESCC presented to our hospital with right side dysfunction of the hand and leg on December 28, 2020. After obtaining a detailed clinical history and reviewing the patient’s medical records, we learned that he was initially admitted to the hospital on May 11, 2019, and diagnosed with ESCC and right adrenal gland metastasis (cT3N1M1, stage IVB, according to the American Joint Committee on Cancer, 8th edition) (8). After that diagnosis, the patient was treated with multiple cycles of chemotherapy alone or combined with immunotherapy; treatments included docetaxel and cisplatin, gemcitabine, and cisplatin combined with nivolumab, and docetaxel and nedaplatin. During these treatment periods, the patient never underwent radiotherapy. After his presentation to our team, he underwent an MRI, which revealed a solitary brain metastasis located in the left side of the parietal lobe; subsequently, gamma knife radiosurgery was performed. Then, a CT scan was performed and showed thickening of the middle esophageal wall (maximum diameter of ~5 cm), swollen lymph nodes in the mediastinum, and a swollen nodule in the right adrenal gland. Laboratory tests showed an alanine aminotransferase level of 233 U/L and an aspartate aminotransferase level of 95 U/L. To exclude liver metastases, an abdominal MRI was performed; the imaging confirmed right adrenal metastasis. The diagnosis was updated to ESCC with mediastinal lymph node, brain, and right adrenal metastases (rT4bN1M1, stage IVB, according to the American Joint Committee on Cancer, 8th edition) (8). Because of the sizeable esophageal tumor and the abnormal liver function, we first administered local therapy instead of systemic therapy. The patient received intensity-modulated radiotherapy (IMRT) of the esophagus and lymph nodes with planning dose fractionation of 60 Gy in 30 fractions, five days a week. After the 26th treatment, however, the patient refused any additional radiotherapy.

**Figure 1:**
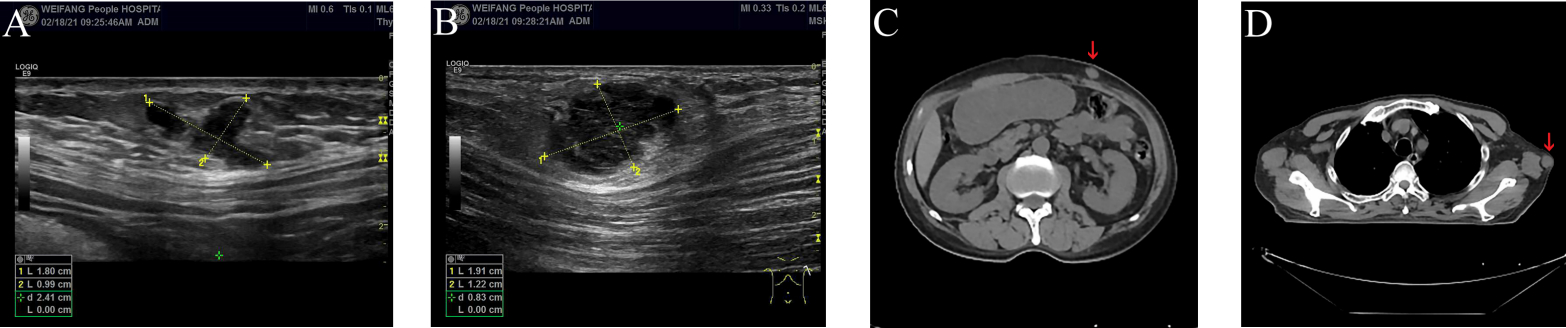
Ultrasound and CT scan of subcutaneous soft tissue metastases **(A–D)**. **(A)** Ultrasound scan of subcutaneous soft tissue metastasis in the left upper quadrant of the abdomen. **(B)** Ultrasound scan of subcutaneous soft tissue metastasis in the left posterior thorax. **(C)** CT scan of subcutaneous soft tissue metastasis in the left upper quadrant of the abdomen (red arrow). **(D)** CT scan of subcutaneous soft tissue metastasis in the left posterior thorax (red arrow).

**Figure 2:**
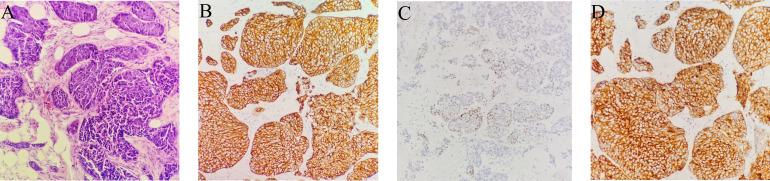
Biopsy of subcutaneous soft tissue metastasis in the left upper quadrant of the abdomen. **(A–D)**. **(A)** Squamous cell carcinoma is observed, which infiltrates the adipose tissue. Atypical epithelioid cells are observed in fibro tissue, muscle tissue and adipose tissue. (hematoxylin and eosin, original magnification × 200). **(B)** Positive CK5/6 immunohistochemical staining in a membranous distribution on the tumor cells (brown) (hematoxylin and eosin, original magnification × 200). **(C)** Positive P40 immunohistochemical staining (brown) (hematoxylin and eosin, original magnification × 200). **(D)** Positive synaptophysin immunohistochemical staining in a membranous distribution on the tumor cells (brown) (hematoxylin and eosin, original magnification × 200).

**Figure 3:**
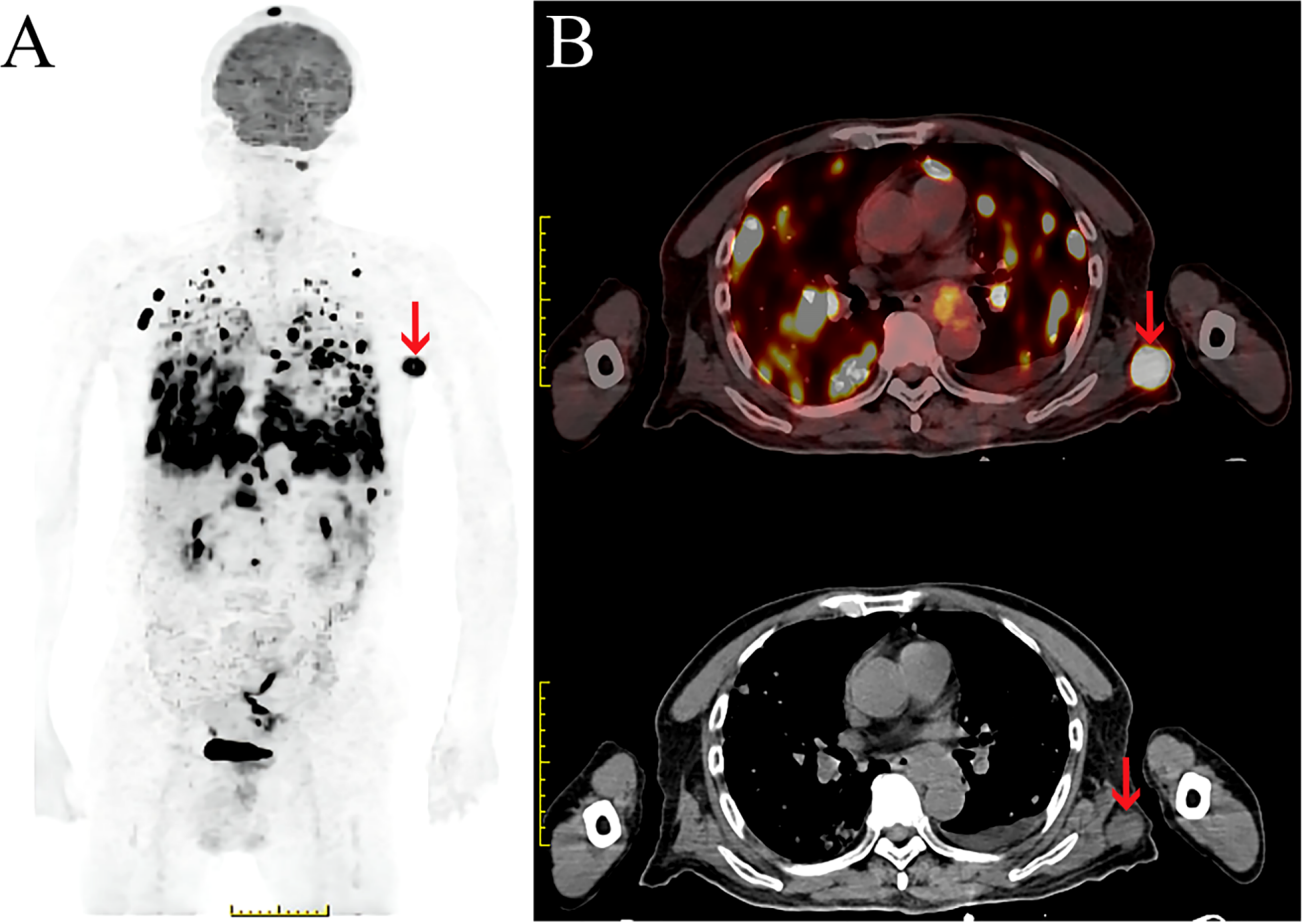
PET/CT scan of subcutaneous soft tissue metastasis in the left posterior thorax. **(A, B)**. **(A)** Maximum intensity projection (MIP) image of the whole body demonstrates a large FDG avid nodule in the left thorax (red arrow). **(B)** Fused PET/CT and CT axial image demonstrates a large FDG avid nodule in the left posterior thorax (SUVmax=20.3) (red arrow).

On February 16, 2021, he was admitted to our hospital again. On physical examination, the patient was noted to have a painless 2-cm anterior abdominal wall swelling and a 2-cm swelling of the left posterior chest wall. The swollen skin did not have an abnormal color. An ultrasound scan demonstrated a 1.8×1.0×1.3 cm lesion along the left upper quadrant of the abdomen and a 1.9×1.2×1.5 cm lesion in the left posterior thorax (**Figures 1A, B**). All the lesions were within the superficial fascia and without dermal involvement. A CT scan confirmed the ultrasound diagnosis (**Figures 1C, D**) and showed a new finding of multiple lung metastases. On excision biopsy, the subcutaneous mass of the left abdomen proved to be metastatic ESCC (**Figure 2A**). The tumor cells were immunohistochemically positive for CK5/6 and P40, compatible with squamous cell carcinoma (**Figures 2B, C**). Moreover, the tumor cells were also positive for synaptophysin, which indicated neuroendocrine differentiation (**Figure 2D**). Because the MRI showed progression of the brain metastases, the patient again underwent gamma knife radiosurgery. The patient had previously received docetaxel combined with cisplatin and gemcitabine combined with cisplatin, so he was treated at this time with camrelizumab (200 mg on day 1) and anlotinib (12 mg on days 2–15). Because of progressive backache, a PET/CT scan was performed in March 10, 2021, to confirm whether other organ metastases had occurred; the scan revealed multiple areas of fluorodeoxyglucose uptake and foci in the esophagus, lung, liver, bone, and right adrenal gland, and in the lymph nodes in the mediastinum, abdomen, and bilateral axillary areas. In addition, an intensely hypermetabolic lesion was localized in the left posterior thorax (SUV_max_=20.3) **(** **Figures 3A, B** **)**. The last time the patient presented to our outpatient service was March 11, 2021, to obtain oxycodone hydrochloride sustained-release tablets; after this time, he was lost to follow-up.

## Discussion

The liver, lungs, bone, and brain are the top four locations for distant metastases of EC (7). Dissemination to subcutaneous soft tissue is a conspicuous rarity. According to a large, retrospective study of 1341 patients with EC, only 25 (1.9%) had metastases to soft tissue, including skeletal muscles and/or subcutaneous fat, among whom seven patients harbored subcutaneous fat metastases (9).

A PubMed search of the English-language literature that reported subcutaneous soft tissue metastases is summarized in **Table 1**. Most patients in these reports had subcutaneous soft tissue metastases located in the chest wall or abdominal wall, which is in accordance with our case report. A large, retrospective study also confirmed this conclusion and suggested that the top four soft tissue metastases are the abdominal wall followed by the back, thigh, and chest wall (16). Soft tissue metastases are rare because of local soft tissue pH, temperature, and metabolite accumulation (9). We speculate that a specific microenvironment probably existed to facilitate the EC cell settlement into the soft tissue; however, the mechanisms driving this process remain uncertain and require clinical and experimental research to determine. Other questions remain unanswered as well. For example, does the overall incidence of subcutaneous soft tissue metastases among ESCC and esophageal adenocarcinoma (EA) differ? A retrospective study showed different overall incidences of soft tissue metastases among the two subtypes, and the incidence of EA was higher. However, this study concentrated on soft tissue metastases instead of subcutaneous soft tissue metastasis specifically; in addition, the incidence of EA is greater than that of ESCC in Western countries, where this study was conducted. Thus, the study may not accurately reflect the global differences between metastases associated with ESCC versus EA.

**Table 1:** Summary of previous studies reporting subcutaneous soft tissue metastases of ESCC.

Authors	Year	Gender/age	Methods of detection	Location	Systemic metastases	Treatment
Smyth et al. (10)	2009	Male/50	CT biopsy	Left buttock	Lung	Radiotherapy
Kapoor et al. (11)	2009	Male/72	PET/CT biopsy	Right posterior chest wall	Lung, bone	NM
Chand et al. (12)	2010	Female/73	US CT biopsy	Left anterior abdomen	Lung, liver, kidneys, and omentum	NM
Balukrishna et al. (13)	2011	Male/56	CT biopsy	Right posterior chest wall	No	Chemotherapy
de Oliveira et al. (14)	2019	Male/41	CT biopsy	Right hemithorax, flank, and armpit	Lymph node of multiple regions, retroperitoneal and pleural nodules	Chemotherapy
Puri et al. (15)	2019	Male/69	CT biopsy	Posterior neck, left index finger, and left abdomen	Lung, liver, and brain	Radiotherapy +chemotherapy

ESCC, esophageal squamous cell carcinoma; NM, not mentioned.

EC does not always contain only one histological component; EC sometimes comprises two or more components and has multidirectional differentiation abilities (17). One hypothesis about ESCC formation is that the subtype develops from totipotential cells at an early age and then transforms with multidirectional differentiation (18). The patient in our case also exhibited neuroendocrine differentiation. We speculate that ESCC with neuroendocrine differentiation has a unique biologic behavior that may facilitate multiple metastases more easily than ESCC without neuroendocrine differentiation.

The treatment of patients with subcutaneous soft tissue metastases must consider oligometastatic or systemic metastases. Patients with oligometastatic EC could benefit from local radiotherapy (19). If the subcutaneous soft tissue metastasis is solitary and no other organs are involved, radical excision may be considered. However, if the soft tissue involvement is combined with systemic metastases, chemotherapy, immunotherapy, and targeted therapy may play more important roles. To our knowledge, available case reports have not discussed immunotherapy in such patients. In recent years, immunotherapy has played a more critical role in the treatment of ESCC and has offered clinicians an alternative when patients develop resistance to chemotherapy (20). According to the ESCORT study, second-line camrelizumab significantly improved overall survival in patients with advanced or metastatic ESCC compared with chemotherapy (21). ALTER1102 showed that the use of anlotinib significantly improved PFS in previously treated, recurrent, or metastatic ESCC patients, who had received one line, two or more lines chemotherapy compared with placebo (22). Recently, a single arm study of anlotinib in combination with PD-1 inhibitors as second-line or later therapy for advanced or metastatic ESCC have obtained a promising result with an objective response rate (ORR) of 30.0% and a disease control rate (DCR) of 87.5% (23). As such, camrelizumab with or without anlotinib may be considered for patients with subcutaneous soft tissue metastasis of ESCC after first-line or later therapy.

## Conclusions

Subcutaneous soft tissue metastases from ESCC are rare. This report highlights the experience of a patient with ESCC that progressed to subcutaneous soft tissue metastases. Clinicians should pay attention to patient symptomatology and administer PET/CT to assist with diagnosis. PD-1 inhibiters with or without anlotinib could be considered as an alternative treatment when metachronous subcutaneous soft tissue metastases occur after first-line chemotherapy or later therapy. Additional study is warranted to investigate the epidemiology, mechanisms, and differences among ESCC and EA with subcutaneous soft tissue metastases. ESCC with neuroendocrine differentiation is a unique subtype of ESCC that warrants more attention from clinicians.

## Data availability statement

The raw data supporting the conclusions of this article will be made available by the authors, without undue reservation.

## Ethics statement

The studies involving human participants were reviewed and approved by Ethics Committee of Weifang People’s Hospital. Written informed consent for participation was not required for this study in accordance with the national legislation and the institutional requirements.

## Author contributions

XG retrieved clinical data, wrote and edited the manuscript. JL and FH supervised the article. HS captured biopsy images and assisted with figure development. ZS captured PET/CT images and assisted with figure development. SQ captured Ultrasound images and assisted with figure development. YL captured CT images and assisted with figure development. YZ assisted with clinical data collection. YC conceive this article, retrieved clinical data, and assisted with editing the manuscript. All authors contributed to the article and approved the submitted version.

## Funding

This work was supported by the Science and Technology Development Project of Weifang City (Grant No. 2020YX008) and the Scientific Research Project of Weifang Municipal Health Commission (Grant No.WFWSJK-2021-163, Grant No.WFWSJK-2021-005).

## Conflict of interest

The authors declare that the research was conducted in the absence of any commercial or financial relationships that could be construed as a potential conflict of interest.

## Publisher’s note

All claims expressed in this article are solely those of the authors and do not necessarily represent those of their affiliated organizations, or those of the publisher, the editors and the reviewers. Any product that may be evaluated in this article, or claim that may be made by its manufacturer, is not guaranteed or endorsed by the publisher.

## References

[1] Sung H Ferlay J Siegel RL Laversanne M Soerjomataram I Jemal A. Global cancer statistics 2020: GLOBOCAN estimates of incidence and mortality worldwide for 36 cancers in 185 countries. CA Cancer J Clin (2021) 71(3):209–49. doi: 10.3322/caac.21660 33538338

[2] Chen WQ Li H Sun KX Zheng RS Zhang SW Zeng HM. [Report of cancer incidence and mortality in China, 2014]. Zhonghua Zhong Liu Za Zhi (2018) 40(1):5–13. doi: 10.3760/cma.j.issn.0253-3766.2018.01.002 29365411

[3] Zhang H-Z Jin G-F Shen H-B. Epidemiologic differences in esophageal cancer between Asian and Western populations. Chin J Cancer (2012) 31(6):281–6. doi: 10.5732/cjc.011.10390 PMC3777490 22507220

[4] Allemani C Matsuda T Di Carlo V Harewood R Matz M Nikšić M. Global surveillance of trends in cancer survival 2000-14 (CONCORD-3): Analysis of individual records for 37 513 025 patients diagnosed with one of 18 cancers from 322 population-based registries in 71 countries. Lancet (2018) 391(10125):1023–75. doi: 10.1016/S0140-6736(17)33326-3 PMC5879496 29395269

[5] Zhao Z Zhang Y Wang X Geng X Zhu L Li M. Clinical response to chemoradiotherapy in esophageal carcinoma is associated with survival and benefit of consolidation chemotherapy. Cancer Med (2020) 9(16):5881–8. doi: 10.1002/cam4.3273 PMC7433822 32627960

[6] Huang T-T Li S-H Chen Y-H Lu H-I Lo C-M Fang F-M. Definitive chemoradiotherapy for clinical T4b esophageal cancer - treatment outcomes, failure patterns, and prognostic factors. Radiother Oncol (2021) 157:56–62. doi: 10.1016/j.radonc.2021.01.007 33482233

[7] Ai D Zhu H Ren W Chen Y Liu Q Deng J. Patterns of distant organ metastases in esophageal cancer: A population-based study. J Thorac Dis (2017) 9(9):3023–30. doi: 10.21037/jtd.2017.08.72 PMC5708381 29221275

[8] Rice TW Ishwaran H Ferguson MK Blackstone EH Goldstraw P. Cancer of the esophagus and esophagogastric junction: An eighth edition staging primer. J Thorac Oncol (2017) 12(1):36–42. doi: 10.1016/j.jtho.2016.10.016 27810391 PMC5591443

[9] El Abiad JM Hales RK Levin AS Morris CD. Soft-tissue metastases from esophageal cancer. J Gastrointest Surg (2019) 23(9):1721–8. doi: 10.1007/s11605-019-04160-w 30809784

[10] Smyth S O’Donnell ME Kumar S Hussain A Cranley B. Atypical presentation of an oesophageal carcinoma with metastases to the left buttock: A case report. Cases J (2009) 2:6691. doi: 10.1186/1757-1626-2-6691 19829843 PMC2740048

[11] Kapoor J Basu S Menon S. Subcutaneous metastasis in esophageal carcinoma detected by FDG-PET imaging. Indian J Cancer (2009) 46(4):354–5. doi: 10.4103/0019-509X.55565 19749476

[12] Chand M Thomas RJ Dabbas N Bateman AC Royle GT. Soft tissue metastases as the first clinical manifestation of squamous cell carcinoma of the esophagus: Case report. World J Oncol (2010) 1(3):135–7. doi: 10.4021/wjon2010.05.209w PMC5649937 29147193

[13] Balukrishna S Jennifer P Viswanathan PN. Solitary subcutaneous metastasis from squamous cell carcinoma of the esophagus: A case report and brief review of literature. J Gastrointest Cancer (2011) 42(4):269–71. doi: 10.1007/s12029-010-9239-8 21174174

[14] de Oliveira RAM da Silva T Piovesana MM Stecca CE Lopes GA Liutti VT. Advanced esophageal neoplasm with subcutaneous metastasis. Case Rep Oncol Med (2019) 2019:9103137. doi: 10.1155/2019/9103137 31179142 PMC6507115

[15] Puri S Holle LM Forouhar FA Clement JM. Subcutaneous metastasis from recurrent basaloid squamous cell carcinoma of the esophagus. J Oncol Pharm Pract (2019) 25(2):492–6. doi: 10.1177/1078155217736920 29078709

[16] Plaza JA Perez-Montiel D Mayerson J Morrison C Suster S. Metastases to soft tissue: a review of 118 cases over a 30-year period. Cancer (2008) 112(1):193–203. doi: 10.1002/cncr.23151 18040999

[17] Yamasaki T Ishii N Okuno T Suekane T Inoue T Nebiki H. A case of esophageal squamous cell carcinoma with neuroendocrine, basaloid, and ciliated glandular differentiation. Clin J Gastroenterol (2021) 14(1):32–8. doi: 10.1007/s12328-020-01267-5 33079336

[18] Nishimaki T Nakagawa S Aizawa K Suzuki T Hatakeyama K Watanabe H. Composite tumor of the esophagus with tripartite differentiation. Dig Dis Sci (1997) 42(5):1041–6. doi: 10.1023/A:1018897321851 9149060

[19] Li B Wang R Zhang T Sun X Jiang C Li W. Development and validation of a nomogram prognostic model for esophageal cancer patients with oligometastases. Sci Rep (2020) 10(1):11259. doi: 10.1038/s41598-020-68160-6 32647289 PMC7347928

[20] Yang J Liu X Cao S Dong X Rao S Cai K. Understanding esophageal cancer: The challenges and opportunities for the next decade. Front Oncol (2020) 10:1727. doi: 10.3389/fonc.2020.01727 33014854 PMC7511760

[21] Huang J Xu J Chen Y Zhuang W Zhang Y Chen Z. Camrelizumab versus investigator’s choice of chemotherapy as second-line therapy for advanced or metastatic oesophageal squamous cell carcinoma (ESCORT): A multicentre, randomised, open-label, phase 3 study. Lancet Oncol (2020) 21(6):832–42. doi: 10.1016/S1470-2045(20)30110-8 32416073

[22] Huang J Xiao J Fang W Lu P Fan Q Shu Y. Anlotinib for previously treated advanced or metastatic esophageal squamous cell carcinoma: A double-blind randomized phase 2 trial. Cancer Med (2021) 10(5):1681–9. doi: 10.1002/cam4.3771 PMC7940231 33586360

[23] Hong Y Wu T Lu P Chang Z Liang W Zhang G. Real-world effectiveness of anlotinib in combination with PD-1 inhibitors as second-line or later therapy for advanced or metastatic esophageal squamous cell carcinoma. J Clin Oncol (2022) 40(4_suppl):320. doi: 10.1200/JCO.2022.40.4_suppl.320 34871037

